# Clinical outcome of laparoscopic vs open right hemicolectomy for colon cancer: A propensity score matching analysis of the Japanese National Clinical Database

**DOI:** 10.1002/ags3.12381

**Published:** 2020-08-01

**Authors:** Takeru Matsuda, Hideki Endo, Masafumi Inomata, Hiroshi Hasegawa, Hiraku Kumamaru, Hiroaki Miyata, Yoshiharu Sakai, Yoshihiro Kakeji, Yuko Kitagawa, Masahiko Watanabe

**Affiliations:** ^1^ The Japan Society for Endoscopic Surgery Tokyo Japan; ^2^ Department of Surgery Kobe University Graduate School of Medicine Kobe Japan; ^3^ Department of Healthcare Quality Assessment Graduate School of Medicine The University of Tokyo Tokyo Japan; ^4^ Department of Gastroenterological and Pediatric Surgery Oita University Faculty of Medicine Yufu City Japan; ^5^ The Japanese Society of Gastroenterological Surgery Tokyo Japan; ^6^ Department of Surgery Graduate School of Medicine Kyoto University Kyoto Japan; ^7^ The Japanese Society of Gastroenterological Surgery Database Committee Tokyo Japan; ^8^ Department of Surgery Keio University School of Medicine Tokyo Japan; ^9^ Department of Surgery Kitasato Institute Hospital Tokyo Japan

**Keywords:** colon cancer, laparoscopic surgery, national database, right hemicolectomy, short‐term outcome

## Abstract

**Aim:**

The advantages of laparoscopic right hemicolectomy over open surgery for colon cancer in general clinical practice are debated, as evidenced by the continued use of open surgery in a significant proportion of patients worldwide. This study aimed to assess and compare the clinical outcome of laparoscopic and open right hemicolectomy for colon cancer using data from the Japanese National Clinical Database.

**Methods:**

A total of 72 299 patients who underwent laparoscopic (n = 46 084) and open (n = 26 215) right hemicolectomy for colon cancer between 2014 and 2018 were enrolled in this retrospective study. Short‐term outcome was compared between groups using propensity score matching analysis.

**Results:**

The incidence of overall postoperative morbidity ≥ Clavien‐Dindo classification grade 3 was significantly higher in the open surgery group than the laparoscopic group (4.7% vs 3.2%, *P* < .001). The incidence of most individual morbidities, including surgical site infection, anastomotic leakage, and ileus, was higher in the open surgery group. Short‐term outcomes, including intraoperative blood loss, postoperative hospital stay, reoperation rate, 30‐day mortality, and in‐hospital mortality, were superior in the laparoscopic group, except for operative time. Subgroup analyses showed that the incidence of postoperative morbidity was lower in the laparoscopic group for all prespecified subgroups.

**Conclusion:**

Laparoscopic right hemicolectomy has an advantage over open surgery for colon cancer with respect to short‐term outcome.

## Graphical abstract

Short‐term outcome of laparoscopic and open right hemicolectomy was analyzed and compared using data of over 70 000 colon cancer patients from the Japanese National Clinical Database. Most morbidities were less frequent and most perioperative outcomes were superior in laparoscopic surgery.

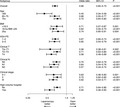

## 1 INTRODUCTION

Since the introduction of laparoscopic surgery for colon cancer in 1991,^1^ its use has been increasing worldwide.^2^ As demonstrated by previous large randomized studies,^3^, ^4^, ^5^, ^6^, ^7^ laparoscopic surgery is now considered one of the standard surgical treatments for colon cancer.^8^, ^9^ However, the previously published data regarding the safety and superiority of laparoscopic surgery were primarily obtained from high‐volume centers or hospitals specializing in colorectal surgery. Therefore, the applicability of their findings to general clinical practice is still being debated.

Right hemicolectomy is one of the most common procedures in colon cancer surgery, as right‐sided cancer accounts for approximately 40% of all colorectal cancers.^10^ However, unlike sigmoidectomy or anterior resection, the laparoscopic approach for this procedure is technically demanding and has not been standardized; there are many variations in technique for lymph node dissection and anastomosis.^11^, ^12^, ^13^, ^14^, ^15^, ^16^, ^17^ This lack of technical standardization may affect surgical outcomes as well as the acceptance of the laparoscopic approach in the general clinical setting. In 2017, only 45.9% of right hemicolectomies were performed laparoscopically in Japan, including benign disease and emergent cases.^18^

To obtain “real world” outcome data of laparoscopic vs open right hemicolectomy for colon cancer, this study conducted a propensity score matching (PSM) analysis of patient data from the Japanese National Clinical Database (NCD).

## 2 MATERIALS AND METHODS

### 2.1 NCD registration

The details of data registration in the Japanese NCD system have been described previously.^19^, ^20^ Briefly, the database began in 2011 as a nationwide registry system linked to the surgical board certification system in Japan. Over 5000 institutions participated in this system in 2018 and approximately 1 400 000 surgical cases are registered annually, corresponding to >95% of all annual surgeries in Japan.

In the gastroenterological section of the NCD, the Japanese Society of Gastroenterological Surgery selected eight main surgical procedures (esophagectomy, distal gastrectomy, total gastrectomy, right hemicolectomy, low anterior resection, hepatectomy, pancreaticoduodenectomy, and surgery for acute diffuse peritonitis) as particularly important in terms of medical standards for surgical quality improvement. All surgical cases registered in the NCD include detailed data regarding morbidity, comorbidity, postoperative complications, and mortality.

### 2.2 Study population

**FIGURE 1:**
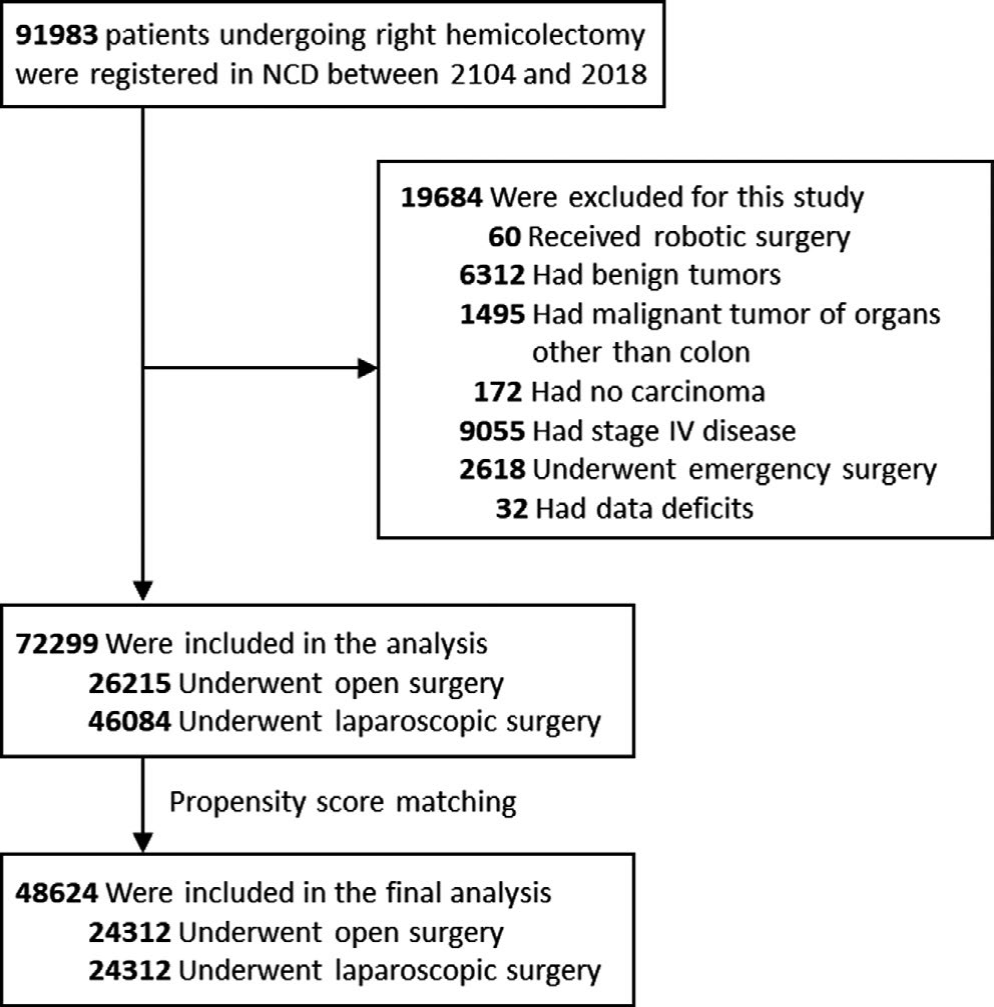
Flowchart diagram illustrating the patient selection process

A total of 91 983 cases of right hemicolectomy were registered in the NCD between January 2014 and December 2018. We excluded robotic surgeries, benign disease, malignant disease of organs other than colon, cT0 disease, cStage IV disease, and emergent surgeries from this study. In addition, 32 cases with data deficits were also excluded. Finally, 72 299 cases of laparoscopic or open right hemicolectomy for colon cancer were enrolled (Figure 1).

### 2.3 Propensity score matching

Propensity score matching between patients who underwent laparoscopic and open right hemicolectomy was conducted to minimize selection bias arising from differences in the study groups' characteristics. Propensity scores were estimated using a multivariable logistic regression model accounting for the following patient parameters: age, sex, body mass index (BMI), American Society of Anesthesiologist physical status (ASA‐PS) score, comorbidities, preoperative blood transfusion, clinical T and N stage according to 7th edition of the American Joint Committee on Cancer TNM classification system,^21^ and history of preoperative chemotherapy. To specifically balance hospital‐level characteristics such as familiarity with the surgical procedure and postoperative management, propensity score estimation was performed separately in two different hospital groups based on annual surgical volume of right hemicolectomy cases, the high‐volume hospital group (≥25 cases per year) and low‐volume hospital group (<25 cases per year). In each group, patients undergoing laparoscopic right hemicolectomy were matched with patients undergoing open surgery at a 1:1 ratio without replacement using a caliper width of 0.2 standard deviation of the logit of the propensity score and finally combined into one study cohort for comparison. The covariate balance achieved by PSM was assessed using standardized mean difference (SMD), which is the most widely used statistic for the assessment of balance after PSM.^22^ An SMD smaller than 0.1 is considered to be well‐balanced.

### 2.4 Study endpoints

The primary endpoint of the study was incidence of overall postoperative morbidity ≥ grade III according to the Clavien‐Dindo (CD) classification. The secondary endpoints included postoperative morbidity ≥ CD grade I, operative time, volume of intraoperative blood loss, incidence of intraoperative transfusion, incidence of conversion to open surgery, reoperation, 30‐day mortality, in‐hospital mortality, length of postoperative hospital stay, and R0 resection rate. Subgroup analyses for overall postoperative morbidity ≥ CD grade III were also performed based on age (<65 vs 65‐75 vs >75 years), ASA‐PS score (1‐2 vs 3‐5), BMI (<18.5 kg/m^2^ vs 18.5‐25 kg/m^2^ vs >25 kg/m^2^), clinical T stage (Tis‐1 vs T2‐3 vs T4), clinical N stage (N0 vs N1 vs N2), clinical stage (stage 0‐I vs stage II vs stage III), and hospital volume (high‐volume vs low‐volume).

### 2.5 Statistical analysis

Pearson's χ^2^ test was used to compare categorical variables. The Wilcoxon rank sum test was used to compare continuous variables. Subgroup analyses were performed using logistic regression and are presented in a forest plot. All statistical tests were two‐sided. *P* < .05 was considered significant. All analyses were conducted using R version 3.6.0 (R Foundation for Statistical Computing).

## 3 RESULTS

Patient and tumor characteristics before and after PSM are shown in Tables 1a and 1b. Before PSM, there were 26 215 and 46 084 patients who underwent open and laparoscopic surgery, respectively. A non‐significant higher proportion of the following variables was present in the open surgery group: age >75 years, ASA‐PS score ≥3, preoperative blood transfusion, and advanced clinical disease stage, including clinical T and N. The proportion of comorbidities, including hypertension, diabetes mellitus, and chronic obstructive pulmonary disease (COPD), and preoperative chemotherapy were similar between the groups. The laparoscopic group had a higher proportion of high BMI (>25 kg/m^2^) patients. After PSM, 24 312 matched pairs were created. All matching covariates were well‐balanced as evidenced by SMD < 0.1.

**TABLE 1a:** Baseline characteristics before and after PSM

	Before PSM			After PSM		
	Open n = 26 215	Laparoscopy n = 46 084	SMD	Open n = 24 312	Laparoscopy n = 24 312	SMD
Age, (%)						
<65	3669 (14.0)	8467 (18.4)	0.195	3545 (14.6)	3705 (15.2)	0.021
65‐75	8633 (32.9)	17 511 (38.0)		8266 (34.0)	8316 (34.2)	
75 <	13 913 (53.1)	20 106 (43.6)		12 501 (51.4)	12 291 (50.6)	
Sex, (%)						
Male	12 571 (48.0)	22 973 (49.9)	0.038	11 770 (48.4)	11 700 (48.1)	0.006
Female	13 644 (52.0)	23 111 (50.1)		12 542 (51.6)	12 612 (51.9)	
ASA‐PS, (%)						
1‐2	21 558 (82.2)	40 226 (87.3)	0.141	20 297 (83.5)	20 490 (84.3)	0.022
3‐5	4657 (17.8)	5858 (12.7)		4015 (16.5)	3822 (15.7)	
BMI, (%)						
<18.5	4980 (19.0)	5306 (11.5)	0.233	4071 (16.7)	4054 (16.7)	0.010
18.5 ≤ BMI < 25	16 749 (63.9)	30 260 (65.7)		15 841 (65.2)	15 947 (65.6)	
25≤	4486 (17.1)	10 518 (22.8)		4400 (18.1)	4311 (17.7)	
Comorbidities						
DM, (%)						
No	21 050 (80.3)	36 824 (79.9)	0.010	19 502 (80.2)	19 557 (80.4)	0.006
Yes	5165 (19.7)	9260 (20.1)		4810 (19.8)	4755 (19.6)	
COPD, (%)						
No	25 356 (96.7)	44 702 (97.0)	0.016	23 535 (96.8)	23 531 (96.8)	0.001
Yes	859 (3.3)	1382 (3.0)		777 (3.2)	781 (3.2)	
Hypertension, (%)						
No	14 982 (57.2)	26 131 (56.7)	0.009	13 884 (57.1)	13 980 (57.5)	0.008
Yes	11 233 (42.8)	19 953 (43.3)		10 428 (42.9)	10 332 (42.5)	
Ischemic heart disease, (%)						
No	25 191 (96.1)	44 435 (96.4)	0.017	23 360 (96.1)	23 417 (96.3)	0.012
Yes	1024 (3.9)	1649 (3.6)		952 (3.9)	895 (3.7)	
Dialysis, (%)						
No	25 989 (99.1)	45 754 (99.3)	0.017	24 117 (99.2)	24 115 (99.2)	0.001
Yes	226 (0.9)	330 (0.7)		195 (0.8)	197 (0.8)	
Cerebrovascular disease, (%)						
No	24 950 (95.2)	44 126 (95.8)	0.028	23 160 (95.3)	23 280 (95.8)	0.024
Yes	1265 (4.8)	1958 (4.2)		1152 (4.7)	1032 (4.2)	
Steroid, (%)						
No	25 959 (99.0)	45 577 (98.9)	0.012	24 069 (99.0)	24 084 (99.1)	0.006
Yes	256 (1.0)	507 (1.1)		243 (1.0)	228 (0.9)	
Bleeding disorder, (%)						
No	25 079 (95.7)	44 153 (95.8)	0.007	23 257 (95.7)	23 242 (95.6)	0.003
Yes	1136 (4.3)	1931 (4.2)		1055 (4.3)	1070 (4.4)	

Abbreviations: ASA‐PS, American Society of Anesthesiologists physical status; BMI, body mass index; COPD, chronic obstructive pulmonary disease; DM, diabetes mellitus; PSM, propensity score matching; SMD, standardized mean difference.

**TABLE 1b:** Baseline characteristics before and after PSM

	Before PSM			After PSM		
	Open n = 26 215	Laparoscopy n = 46 084	SMD	Open n = 24 312	Laparoscopy n = 24 312	SMD
Preoperative blood transfusion, (%)						
No	24 817 (94.7)	45 186 (98.1)	0.181	23 454 (96.5)	23 503 (96.7)	0.011
Yes	1398 (5.3)	898 (1.9)		858 (3.5)	809 (3.3)	
Preoperative chemotherapy, (%)						
No	25 951 (99.0)	45 838 (99.5)	0.054	24 124 (99.2)	24 106 (99.2)	0.008
Yes	264 (1.0)	246 (0.5)		188 (0.8)	206 (0.8)	
Clinical T^a^, (%)						
T1	1395 (5.3)	7403 (16.1)	0.507	1395 (5.7)	1305 (5.4)	0.024
T2	2186 (8.3)	6564 (14.2)		2181 (9.0)	2224 (9.1)	
T3	14 788 (56.4)	22 686 (49.2)		14 214 (58.5)	14 382 (59.2)	
T4a	5212 (19.9)	6281 (13.6)		4692 (19.3)	4661 (19.2)	
T4b	2191 (8.4)	1392 (3.0)		1405 (5.8)	1322 (5.4)	
Tis	397 (1.5)	1717 (3.7)		396 (1.6)	388 (1.6)	
TX	46 (0.2)	41 (0.1)		29 (0.1)	30 (0.1)	
Clinical N^a^, (%)						
N0	14 219 (54.2)	29 714 (64.5)	0.235	13 551 (55.7)	13 535 (55.7)	0.013
N1a	4237 (16.2)	6687 (14.5)		3953 (16.3)	3982 (16.4)	
N1b	3425 (13.1)	4792 (10.4)		3133 (12.9)	3145 (12.9)	
N1c	80 (0.3)	103 (0.2)		70 (0.3)	69 (0.3)	
N2a	2721 (10.4)	3274 (7.1)		2401 (9.9)	2378 (9.8)	
N2b	1404 (5.4)	1474 (3.2)		1154 (4.7)	1165 (4.8)	
NX	129 (0.5)	40 (0.1)		50 (0.2)	38 (0.2)	
Clinical stage^a^, (%)						
Stage 0	393 (1.5)	1706 (3.7)	0.444	393 (1.6)	383 (1.6)	0.011
Stage I	2977 (11.4)	12 364 (26.8)		2976 (12.2)	3018 (12.4)	
Stage II	10 833 (41.3)	15 621 (33.9)		10 167 (41.8)	10 118 (41.6)	
Stage III	11 867 (45.3)	16 330 (35.4)		10 711 (44.1)	10 739 (44.2)	
Stage X	145 (0.6)	63 (0.1)		65 (0.3)	54 (0.2)	
High‐volume hospital, (%)						
No	18 891 (72.1)	26 247 (57.0)	0.320	17 087 (70.3)	17 087 (70.3)	<0.001
Yes	7324 (27.9)	19 837 (43.0)		7225 (29.7)	7225 (29.7)	

Abbreviations: PSM, propensity score matching; SMD, standardized mean difference.

^a^ Tumors were classified according to the 7th edition of the American Joint Committee on Cancer TNM classification.

Surgical outcomes are shown in Table 2. The incidence of overall postoperative morbidity ≥ CD grade III was significantly higher in the open surgery group than the laparoscopic group (4.7% vs 3.2%, *P* < .001). The individual incidence of superficial surgical site infection (SSI), deep SSI, intra‐abdominal abscess, anastomotic leakage, pneumonia, deep vein thrombosis, paralytic ileus, and adhesive ileus was also significantly higher in the open surgery group. The incidence of pulmonary embolism, intra‐abdominal bleeding, and severe postoperative ascites did not differ `significantly between the groups.

**TABLE 2:** Comparison of short‐term outcomes after PSM

	Open n = 24 312	Laparoscopy n = 24 312	*P*
Overall postoperative morbidity (≥CD III), (%)	1153 (4.7)	775 (3.2)	<.001
Overall postoperative morbidity (≥CD I), (%)	5977 (24.6)	4272 (17.6)	<.001
Superficial SSI, (%)	1311 (5.4)	658 (2.7)	<.001
Deep SSI, (%)	369 (1.5)	123 (0.5)	<.001
Intra‐abdominal abscess, (%)	403 (1.7)	238 (1.0)	<.001
Anastomotic leakage, (%)	371 (1.5)	234 (1.0)	<.001
Pneumonia, (%)	333 (1.4)	224 (0.9)	<.001
Pulmonary embolism, (%)	29 (0.1)	22 (0.1)	.401
Deep vein thrombosis, (%)	86 (0.4)	61 (0.3)	.047
Intra‐abdominal bleeding, (%)	24 (0.1)	27 (0.1)	.779
Paralytic ileus, (%)	718 (3.0)	543 (2.2)	<.001
Adhesive ileus, (%)	182 (0.7)	117 (0.5)	<.001
Severe ascites, (%)	30 (0.1)	20 (0.1)	.203
Re‐operation within 30 d (%)	667 (2.7)	441 (1.8)	<.001
Mortality within 30 d, (%)	131 (0.5)	61 (0.3)	<.001
In‐hospital mortality, (%)	233 (1.0)	110 (0.5)	<.001
Postoperative hospital stay^a^, d (IQR)	14 (11‐21)	11. (9‐15)	<.001
Operative time^a^, min (IQR)	160 (125‐203)	216 (175‐266)	<.001
Estimated blood loss^a^, mL (IQR)	100 (40‐211)	30 (10‐80)	<.001
Intraoperative transfusion, (%)	2172 (8.9)	1383 (5.7)	<.001
Resection status, (%)			
R0	23 608 (97.1)	23 896 (98.3)	<.001
R1	331 (1.4)	216 (0.9)	
R2	191 (0.8)	86 (0.4)	
Rx	182 (0.7)	114 (0.5)	
Conversion to open surgery, (%)	NA	1043 (4.3)	NA

Abbreviations: CD, Clavien‐Dindo classification; IQR, interquartile range; SSI, surgical site infection.

^a^ The data are expressed as the median (IQR).

Although the operative time was significantly shorter in the open group (160 vs 216 minutes, *P* < .001), the open group had a significantly greater volume of intraoperative blood loss (100 vs 30 mL, *P* < .001) and frequency of intraoperative blood transfusion (8.9% vs 5.7%, *P* < .001). The length of postoperative hospital stay was also significantly longer in the open surgery group (14 vs 11 days, *P* < .001). Rate of reoperation (2.7% vs 1.8%, *P* < .001), 30‐day mortality (0.5% vs 0.3%, *P* < .001), and in‐hospital mortality (1.0% vs 0.5%, *P* < .001) were significantly higher in the open surgery group. Curative resection (R0 resection) was achieved more frequently in the laparoscopic group (98.3% vs 97.1%, *P* < .001). The rate of conversion from laparoscopic to open surgery was 4.3%.

**FIGURE 2:**
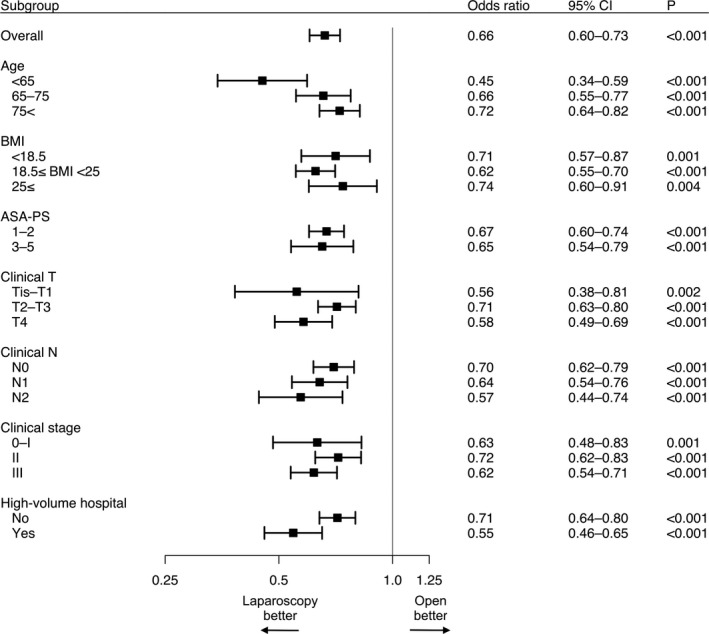
Forest plot on the association of operative approach with overall postoperative morbidity ≥ Clavien–Dindo classification grade III for different subgroups. CI, confidence interval

Forest plot on the association of operative approach with overall postoperative morbidity ≥ CD grade III for different subgroups is shown in Figure 2. Laparoscopic surgery decreased the risk of postoperative morbidity in all different subgroups.

## 4 DISCUSSION

Although previous large‐scale clinical trials have demonstrated the superiority of laparoscopic surgery for colon cancer to open surgery with respect to short‐term and long‐term outcomes, debate continues regarding its applicability in general clinical practice. The debate over right hemicolectomy seems particularly reasonable because of its technical difficulty and various techniques. Few large studies have reported outcomes of laparoscopic right hemicolectomy using data from sources other than clinical trials.^23^, ^24^, ^25^ To our knowledge, the present study is the largest to compare outcomes of laparoscopic vs open right hemicolectomy for colon cancer using “real world” data.

In this study, the incidence of most all postoperative complications was significantly reduced in laparoscopic surgery compared with open surgery except for pulmonary embolism, intra‐abdominal bleeding, and severe ascites. In contrast, Jurowich et al^23^ reported no relevant advantage for laparoscopic right hemicolectomy for colon cancer based on a study of patient data from the DGAV StuDoQ|ColonCancer registry in Germany. Although they performed a propensity score analysis, >80% of patients received open surgery overall. In addition, the rate of conversion from laparoscopic to open surgery was 16.5% in their study, considerably higher than our 4.3% conversion rate, although the reason for conversion was not available from the Japanese NCD. Interestingly, JCOG0404, a randomized controlled trial conducted by the Colorectal Cancer Study Group of the Japan Clinical Oncology Group (JCOG) to confirm the non‐inferiority of laparoscopic surgery compared with open surgery for patients with stage II/III colon cancer, also did not demonstrate the significant differences between the laparoscopic and open surgery in terms of anastomotic leakage, paralytic ileus, and adhesive ileus.^26^ One possible reason for the different outcomes between JCOG0404 and the present study is the eligibility criteria of the study. The present study included right hemicolectomy alone, while right hemicolectomy accounted for only 21% and 19% of laparoscopic and open surgeries, respectively, in JCOG0404. Another reason is the study scale. In the present study, the difference reached statistical significance despite the relatively small difference in the value, because the study scale was sufficiently large. On the other hand, a population‐based study using the Premier Healthcare Database in the United States reported a significantly lower incidence of anastomotic leakage, bleeding, and infection in minimally invasive right colectomy compared with open surgery; however, benign disease, cecectomy, and robotic surgery were included in that data.^25^ The indications and acceptance of the laparoscopic approach for colon cancer seem to vary according to locality or country. Our data based on the Japanese NCD strongly support superior short‐term outcomes for laparoscopic right hemicolectomy as long as high surgical quality is assured.

To obtain reliable and valid study results, validation of surgical quality is essential. In the present study, hospital surgical volume was defined according to the number of annual surgical cases and employed for stratification prior to PSM to minimize potential bias. Approximately 22 000 cases of right hemicolectomy from over 4200 hospitals were registered yearly in the NCD system from 2014 to 2018, including stage IV disease and emergent surgeries.^18^ The median number of right hemicolectomies per hospital was eight or nine every year (the minimum number was one and the maximum was 92 to 124, data not shown). Using a cut‐off number of ≥25 cases per year to define a high‐volume hospital as in this study, approximately 13% of the NCD‐participating hospitals would qualify as high‐volume and 37% of all right hemicolectomies in Japan were performed in those high‐volume hospitals.

As expected, patients in this study with more advanced disease and higher ASA‐PS score tended to receive open surgery rather than laparoscopic surgery. On the other hand, all prespecified comorbidities, such as COPD and ischemic heart disease, were well‐balanced between the groups before PSM. Patients with higher BMI or preoperative chemotherapy tended to undergo laparoscopic surgery rather than open surgery. These results affirm the feasibility and safety of the laparoscopic approach for high‐risk patients with severe comorbidities undergoing colorectal surgery shown in previous studies.^27^, ^28^, ^29^ The results of the subgroup analyses also support the applicability of the laparoscopic approach to colon cancer patients in various conditions.

The present study has several limitations. First, it is a retrospective observational study. A potential bias due to heterogeneity of surgical quality or hospital performance cannot be excluded. However, it was reduced to a minimum by employing PSM stratified by hospital volume. Second, oncological and long‐term outcomes are not available from the Japanese NCD. Only data regarding baseline characteristics and short‐term outcomes are entered in the Japanese NCD, as well as the American College of Surgeons' National Surgical Quality Improvement Program. A definitive conclusion regarding the oncological validity of laparoscopic right hemicolectomy would require data from elsewhere.

In conclusion, this propensity score‐matched study using a nationwide Japanese database showed the superiority of laparoscopic right hemicolectomy over open surgery for colon cancer with respect to all short‐term outcomes except for operative time. These advantages seem applicable to most colon cancer patients regardless of comorbidities or condition. However, confirming the oncological outcome of this surgery by other sources is also important.

## CONFLICT OF INTEREST

Hideki Endo, Hiraku Kumamaru, and Hiroaki Miyata are affiliated with the Department of Healthcare Quality Assessment at the University of Tokyo, which is a social collaboration department supported by grants from the National Clinical Database, Johnson & Johnson KK, and Nipro Co. Yuko Kitagawa has received research grants from Chugai Pharmaceutical Co., Ltd. and Taiho Pharmaceutical Co., LTD. The other authors have no conflicts of interest.

## ETHICAL APPROVAL

The study protocol was approved by the institutional review board of Kobe University (approval number B190247).
